# Five children with haploinsufficiency of A20 caused by heterozygous mutations in the *TNFAIP3* gene

**DOI:** 10.3389/fimmu.2026.1738656

**Published:** 2026-03-11

**Authors:** Hengpan Yao, Yijing Liu, MengJun Dong, Kairui Yang, Zhidan Yu, Fang Zhou

**Affiliations:** 1 Department of Digestion, Children's Hospital Affiliated to Zhengzhou University, Henan Children’s Hospital Zhengzhou Children’s Hospital, Zhengzhou, Henan, China; 2 Zhengzhou Key Laboratory of Children’s Digestive Diseases, Children’s Hospital Affiliated to Zhengzhou University, Zhengzhou, Henan, China

**Keywords:** A20, autoinflammatory diseases, haploinsufficiency, heterozygous variants, pediatric, TNFAIP3

## Abstract

**Objectives:**

Children with A20 haploinsufficiency, resulting from heterozygous variants in the *TNFAIP3* gene, are increasingly being identified. However, their diagnosis and treatment remain challenging and are not yet fully optimized. The clinical, genetic characteristics and treatment methods of five children with HA20 from different families were collected from Henan Children’s Hospital between April 2019 and August 2023 to evaluated for accumulating experience in the management of this rare condition.

**Results:**

We identified five heterozygous variants in the *TNFAIP3* gene among the five children, including c.866delA: p.H289Pfs* 3, c.1243_1247delAAAAC: p. N416Tfs* 11, NC_000006.11: g.136693638_138817508del, c.133C>T: p.R45X, c.1903_1906delAAAC: p. K635fs* 61. All patients were *de novo* (they have all been tested to confirm their origin). Besides, patient 3 also harbored two *MMACHC* gene mutations: c.349G>C: p.A117P-–inherited from the parents and c.482G>A: p.R161Q -–*de novo*. Variants in patients 3 and 5 have not been reported. All five patients presented with childhood-onset recurrent fever and intermittent diarrhea, which are hallmark features of HA20. Additionally, two of the five patients experienced intermittent bloody stool, three had oral ulcers, and two presented with skin symptoms, further aligning with the clinical manifestations of HA20. Laboratory tests revealed elevated inflammatory markers, including increased white blood cell (WBC) counts, C-reactive protein (CRP), and erythrocyte sedimentation rate (ESR). Endoscopic observation, there were ulcers in different parts of the intestine. Each child was treated with the oral drug thalidomide, 4 children (80%) received glucocorticoids to reduce inflammation, and had different biological agents according to individual differences. During follow-up, we observed significant improvement in all children who received targeted treatment.

**Conclusions:**

HA20 is a rare monogenic early-onset auto-inflammatory disease. It can present with a variety of clinical manifestations, including Behcet disease, inflammatory bowel disease, lupus-like syndrome and periodic fever syndrome. Whole-exome sequencing should be actively considered for children who present with early-onset symptoms or features suggestive of autoimmune diseases.

## Introduction

HA20 (Haploinsufficiency of A20) was first reported in 2016 by Zhou et al (1). To date, over 100 cases have been documented, predominantly among Japanese populations, with relatively fewer reports from China. HA20 is an autosomal dominant hereditary disorder caused by pathogenic variants in the tumor necrosis factor (TNF)-α-induced protein 3 gene (*TNFAIP3*) (2–4). The A20 protein, encoded by *TNFAIP3*, contains two functional domains: the N-terminal OTU domain and the C-terminal ZnF (zinc finger) domain, both of which play critical roles in regulating the nuclear factor κB (NF-κB) signaling pathway. Specifically, A20 facilitates the degradation of activated NF-κB through ubiquitination, thereby inhibiting inflammatory responses. In HA20 patients, variants in *TNFAIP3* typically result in loss-of-function of the A20 protein, leading to dysregulation of the NF-κB pathway and subsequent recurrent autoinflammatory responses. These variants often manifest as nonsense variants, causing premature termination of A20 protein translation and production of a truncated, non-functional protein. Clinical manifestations of HA20 are diverse and include recurrent fever, diarrhea, oral and genital ulcers, uveitis, and vasculitis (5–11). When these symptoms appear, especially in pediatric patients, HA20 should be considered as a differential diagnosis. Early-stage HA20 often presents with recurrent mucosal ulcers, necessitating differentiation from conditions such as rheumatoid arthritis, juvenile idiopathic arthritis, periodic fever with aphthous stomatitis, pharyngitis, and adenitis (PFAPA), hyperimmunoglobulinemia Dsyndrome (HIDS), and inflammatory bowel disease. Whole-exome sequencing (WES) aids clinicians in identifying *TNFAIP3* variants, thus confirming the diagnosis of HA20.

Several studies highlight that clinical presentations of HA20 can vary even among patients with identical variants or within the same family. Moreover, patients with the same pathogenic variant may respond differently to the same treatment regimen, suggesting that other genetic or environmental factors may influence HA20 pathogenesis. Currently, there is no standardized treatment approach for HA20. While most children respond well to glucocorticoids, long-term use is associated with significant side effects. Colchicine therapy has shown efficacy in mild to moderate cases. Treatment selection appears to depend on the patient’s primary clinical phenotype. Responses to immunosuppressive agents (methotrexate, cyclosporine, hydroxychloroquine, thalidomide, tacrolimus, and malacenthol) have been variable. For refractory cases, biologics (TNF-α inhibitors, anti-IL-1 drugs, anti-IL-6 drugs, and JAK inhibitors) offer potential new treatment options (12, 13). A minority of patients have achieved remission through hematopoietic stem cell transplantation (HSCT), including both autologous and allogeneic transplants. Given the limited number of HA20 studies, clear diagnostic criteria have yet to be established. Gene sequencing combined with clinical manifestations can assist in diagnosis. This study analyzed the clinical and genetic data of five unrelated Chinese families to enhance understanding of HA20 diagnosis and treatment.

## Methods

A retrospective analysis was conducted on five children diagnosed with HA20 at Henan Children’s Hospital between April 2019 and August 2023. Informed consent was obtained from all legal guardians to review the electronic medical records of the diagnosed children. Genetic testing data from whole-exome sequencing (WES), along with demographic, clinical, and laboratory data, were collected for each child. We also performed sanger sequencing (**Supplementary Material 1**). Imaging results, including ultrasound and magnetic resonance imaging, and gastrointestinal findings were also reviewed for some patients. The course of illness, symptom exacerbation and relief, targeted drug usage (corticosteroids, infliximab, adalimumab, etc.), symptom changes, and treatment outcomes post-discharge were recorded.

## Case series

### Demographic and clinical characteristics

Five children with HA20 were confirmed in the Gastroenterology Department of Henan Children’s Hospital between April 2019 and August 2023. **Table 1** summarizes their baseline demographics and clinical characteristics and treatment follow-up. All patients aged from 2 to 10 years and experienced childhood-onset symptoms, with two female and three male patients. None of the patients reported a family history of genetic disorders or any special allergies. All five children with HA20 exhibited common symptoms of recurrent fever and gastrointestinal symptoms, including abdominal pain, diarrhea and gastrointestinal ulcers at the onset of the disease. Oral ulcers were observed in three patients (60%), skin lesions on the extremities and trunk in two patients (40%), genital ulcers in one patient (20%), arthritis in one patient (20%), motor and cognitive development delay in two patients (40%). In routine blood tests, inflammatory markers (CRP, ESR) were elevated in all five children. Notably, CRP levels were particularly high in cases 2, 3 and 4, while significant hematological sedimentation was observed in cases 1, 3 and 5. Hemoglobin values were low in all patients which was considered to be related to inflammation (**Table 2**).

**Table 1:** Characteristics of patients with *TNFAIP3* variants.

Characteristics	Patient 1	Patient 2	Patient 3	Patient 4	Patient 5
Genomic location (GRCh37/ hg19)	c.866delA: p.H289Pfs* 3	c.1243_1247delAAAAC: p. N416Tfs* 11	NC_000006.11: g.136693638_138817508del	c.133C>T: p. R45X	c.1903_1906delAAAC: p. K635fs* 61
Sex	F	M	M	F	M
Age at genetic diagnosis (years)	5y8m	6m	2y7m	10m	1y6m
Recurrent fever	YES	YES	YES	YES	YES
Oral ulcers	YES	NO	YES	NO	YES
Genital ulcers	NO	NO	YES	NO	NO
Skin lesions	NO	YES	YES	NO	NO
Gastrointestinal lesions	YES	YES	YES	YES	YES
Arthritis	NO	NO	YES	NO	NO
Motor and cognitive development delay	YES	NO	NO	NO	YES
Treatment	Thalidomide, glucocorticoid, infliximab, adalimumab	Thalidomide, glucocorticoid	Thalidomide, glucocorticoid	Thalidomide	Thalidomide, glucocorticoid
Follow-up	2y3m; There is an ulcer in the ileocecal region	3y; Symptoms and endoscopic findings were completely relieved	1y; Symptoms and endoscopic findings were completely relieved	3y4m; Symptoms and endoscopic findings were completely relieved	1m; Symptoms were completely relieved

a F: Female; M: Male; y: year; m: month; *TNFAIP3* transcript: NM_006290.5.

**Table 2:** Laboratory findings of HA20 patients.

Laboratory findings	Patient 1	Patient 2	Patient 3	Patient 4	Patient 5
WBC (5~12 × 109/L)	13.45	20.2	18.45	13.41	10.98
Hemoglobin (120~140 g/L)	90	110	91	94.2	103
Platelets (100~300 × 109/L)	303	221	378	454	304
CRP (0~10.0 mg/L)	15.5	43.91	99.19	108.54	12.25
ESR (0~15 mm/h)	68	27	46	19	86
IL-6 (0-7pg/mL)	35.99	39.6	/	/	19.76
Autoantibodies	Negative	ANA (1∶100)	Negative	/	/

### Characteristics of *TNFAIP3* mutation

**Figure 1:**
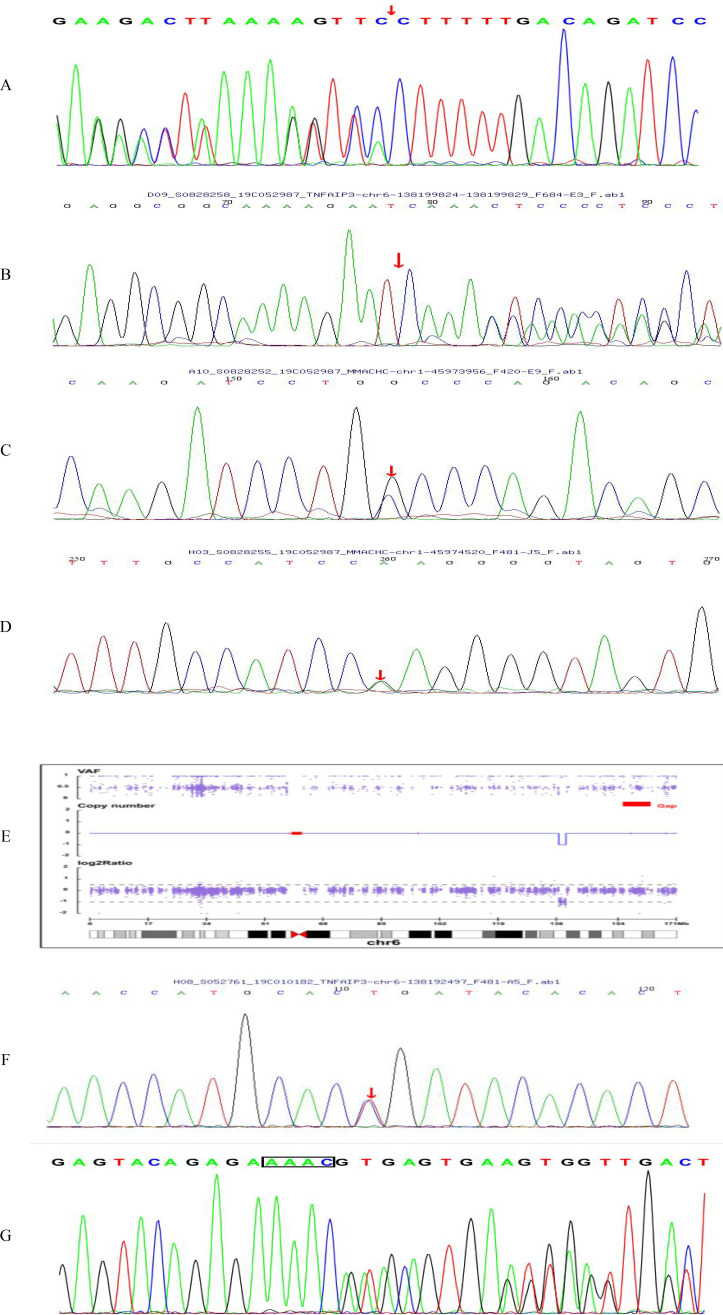
Electropherogram and CNV plot. **(A)** Electropherogram of patient 1 showing c.866delA: p.H289Pfs* 3 variant. **(B)** Electropherogram of patient 2 showing c.1243_1247delAAAAC: p. N416Tfs* 11 variant in *TNFAIP3*. **(C, D)** Electropherogram of patient 2 showing c.349G>C: p.A117P variant and c.482G>A: p.R161Q variant in *MMACHC*. **(E)** CNV plot of patient 3 showing NC*000006.11: g.136693638*138817508del variant. **(F)** Electropherogram of patient 4 showing c.133C>T: p.R45X variant. **(G)** Electropherogram of patient 5 showing c.1903_1906delAAAC: p. K635fs* 61 variant.

Genetic confirmation experiments show that heterozygous variants in the *TNFAIP3* gene among the five patients (**Figure 1**). Specifically, Patient 4 exhibited a nonsense mutation characterized by a base substitution of c.133C>T, resulting in an amino acid sequence alteration of p.R45X. Patient 3 presented with copy number variation, detailed as NC_000006.11:g.136693638_138817508del. Patients 1, patient 2 and patient 5 showed small indels including c.866delA: p.H289Pfs* 3, c.1243_1247delAAAAC: p.N416Tfs* 11, c.1903_1906delAAAC: p.K635fs* 61. Further analysis indicated that the variants in all patients were *de novo*. Variants in patients 3 and 5 have not been reported. Variants NC_000006.11: g.136693638_138817508del (PVS1_Very strong+PS4_Moderate + PM2 Supporting) and c.1903_1906del AAAC: p. K635fs* 61 (PVS1_Very strong + PS2_Moderate + PM2_Supporting) were classified as pathogenic per ACMG criteria.

### Endoscopic characteristics

**Figure 2:**
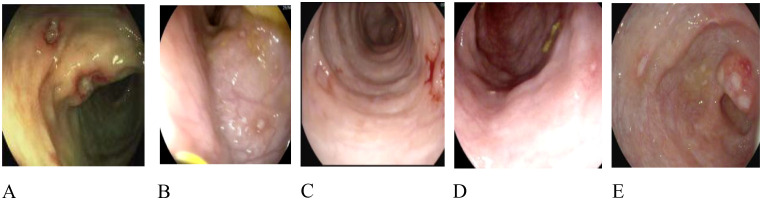
Endoscopic characteristics. **(A)** Multiple ulcers in the descending colon in patient 1. **(B)** Multiple ulcers in the ascending colon in patient 2. **(C)** Multiple ulcers in the transverse colon in patient 3. **(D)** Multiple ulcers in the descending colon in patient 4. **(E)** Multiple ulcers in the ileumecal valve in patient 5.

Gastrointestinal ulcers are a common issue in children with HA20, varying in location and severity (**Figure 2**). Specifically, patients 1, 2, and 3 exhibited multiple ulcers in the colon and rectum. Patient 4 had ulcers in the transverse colon, descending colon, sigmoid colon, and rectum. Patient 5 presented with ulcers in the ileocecum, ascending colon, transverse colon, and descending colon.

### Treatment and follow-up

Patient 1 developed severe hormone-dependent allergic reactions during treatment with glucocorticoids, thalidomide, and infliximab. Symptoms improved after switching to adalimumab. Patients 2, 3, and 5 were treated with corticosteroids and thalidomide. Patient 4 underwent surgical repair for jejunal perforation and was subsequently treated with oral thalidomide. Additionally, patient 2 was diagnosed with HA20 and methylmalonic aciduria (MMA) and received hydroxocobalamin (1 mg daily). After one week, urine organic acid screening and blood homocysteine levels normalized, and hydroxocobalamin dosage was tapered to 1.5 mg weekly. Patients 4, 2, and 3 were followed up for 3 years and 4 months, 3 years, and 1 year, respectively, with complete resolution of symptoms. Patient 1 was followed for 2 years and 3 months, showing improvement in digestive tract symptoms but persistent cecal ulcers. Patient 5 experienced improvement in gastrointestinal symptoms after one month.

## Discussion

**Figure 3:**
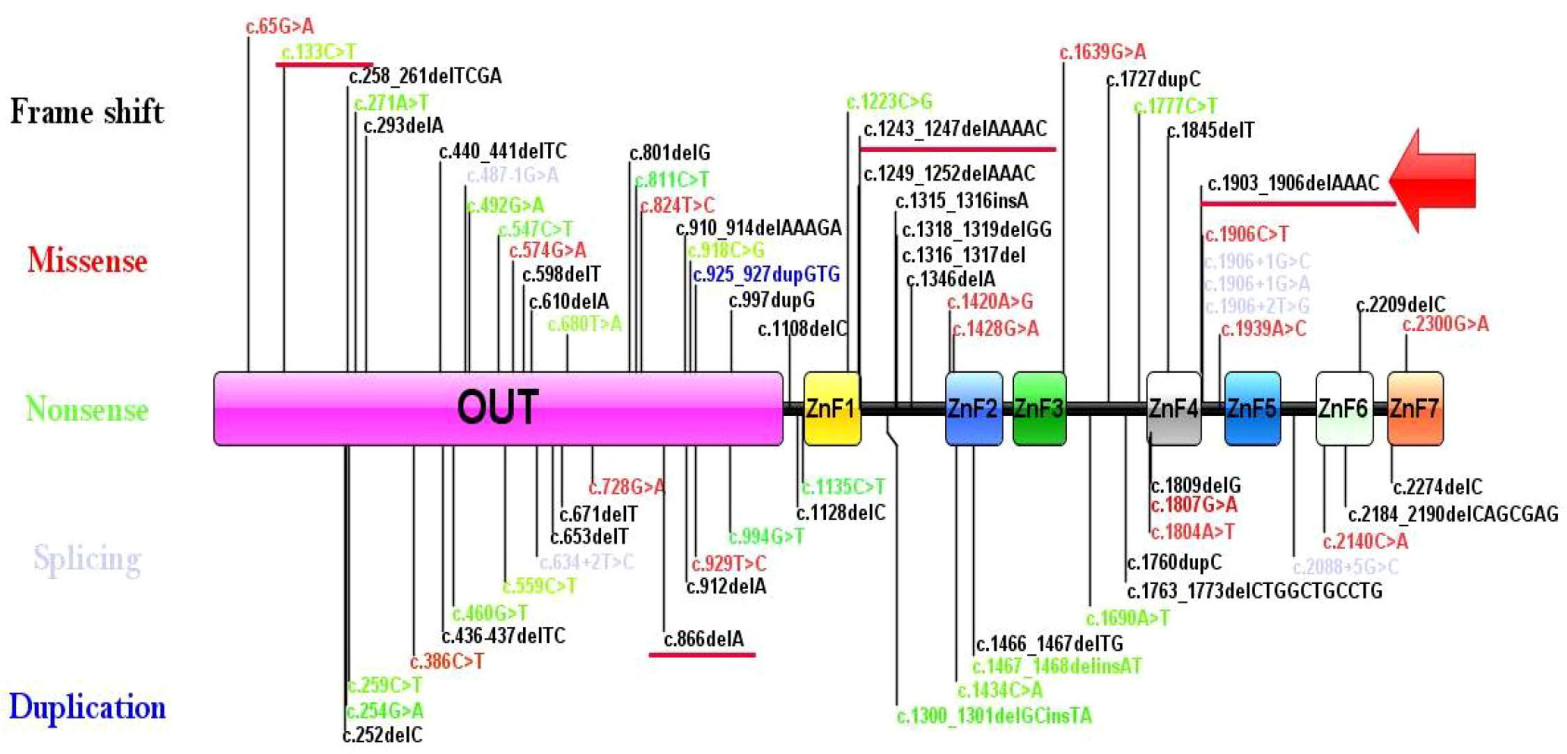
The locations of variants in the *TNFAIP3* gene. The black represents the frameshift variant. The red represents the missense variant. The green represents the nonsense variant. The purple represents the splicing variant. The blue represents the duplication variant. The red arrow represents the new variant. The red underline represents the mutations reported in this article.

We conducted a systematic literature review of articles reporting *TNFAIP3* variants from 2016 to January 2024 (14). A total of 168 patients from 85 families with genetically confirmed HA20 were included, excluding those with additional gene variants or incomplete data. The cohort comprised 61 males and 101 females, with the earliest onset at five days and the longest disease duration exceeding 60 years. Mutation sites in the *TNFAIP3* gene varied widely (**Figure 3**) (15, 16). The *TNFAIP3* gene encodes the A20 protein, which plays a crucial role in regulating NF-κB signaling. Aberrant activation of NF-κB signaling is closely associated with multiple autoimmune diseases (17–19). Therefore, these variants may impair A20 protein function, affecting NF-κB regulation and increasing the risk of autoimmune diseases (20–22). Notably, despite all patients having heterozygous *TNFAIP3* variants, clinical manifestations varied, likely due to differences in mutation type, location, and genetic background. For instance, segmental deletions in patient 3 may affect a broader region of the gene with more complex physiological effects, while spontaneous variants in patients 1, 2, 4, and 5 may result in varying degrees of impaired A20 function. Future studies will explore how these variants influence clinical presentation and treatment response, aiding in understanding disease pathogenesis and improving treatment options.

Clinical phenotypic differences in HA20 are not solely determined by genetic mutation sites. A20 is a highly conserved protein with two domains: the amino-terminal OTU domain and seven carboxyl-terminal ZnF domains (23–25). Patients with impaired OTU or ZnF domains alone do not develop ocular dysfunction, atrophic gastritis, or dental abnormalities. Specifically, OTU domain variants do not cause musculoskeletal disease, autoimmune thyroid disease, liver injury, recurrent respiratory infections, or interstitial lung disease, while ZnF domain variants do not lead to kidney injury or other symptom variations. Thus, clinical phenotype variation may be influenced by modifier genes and/or environmental factors. Variants in ZnF domains correlate with earlier HA20 onset compared to OTU domain variants. Studies show that patients in the OTU group (median onset age: 10 years, IQR: 8–14) have later onset than those in the ZnF group (median onset age: 2.5 years, IQR: 0.6–5). In our study, two *TNFAIP3* variants affected the ZnF domain (patients 2, 5), with onset ages of 6 months and 1 year 6 months, consistent with Chen’s study. Two variants affected the OTU domain (patients 1, 4), with onset ages of five years and shortly after birth. Variant in patient 3 affects the entire gene, with onset age of 1 year 7 months. The small sample size in this study led to discrepancies with the median onset age of the OTU group in Chen’s study. Chen also observed diverse clinical manifestations in HA20 patients evaluated for his study (26). Specifically, the most common symptom was oral ulcers (70%), followed by recurrent fever (42%), gastrointestinal ulcers (40%), skin lesions (38%), genital ulcers (36%), and musculoskeletal disorders (34%). In contrast, in this report, recurrent fever was the most frequent symptom (100%), followed by gastrointestinal ulcers (80%), oral ulcers (60%), skin lesions (40%), motor and cognitive development delay (40%), musculoskeletal disorders (20%), and genital ulcers (20%). These discrepancies from previous literature may be attributed to the small sample size.

HA20 must also be differentiated from other diseases such as Behcet disease (BS) (27–29). Many clinical features of HA20 are similar to BS, including oral ulcers, genital ulcers, gastrointestinal ulcers, and skin lesions (28, 30), leading to initial misdiagnosis. For instance, patient 1 and patient 4 exhibited gastrointestinal ulcers; patient 2 had rash, gastrointestinal ulcers, and oral ulcers; patient 3 presented with rash, gastrointestinal ulcers, and perianal ulcers, all of which can be easily confused with BS. The median age at onset for HA20 patients was 5.92 years (IQR: 1-10), typically occurring in early childhood, whereas BS usually manifests in early adulthood. Recurrent fever, severe intestinal inflammation, elevated acute phase reactants, and fluctuations in autoantibodies are less common in BS patients (31). Infantile-onset inflammatory bowel disease (IBD) with perianal lesions is a form of monogenic IBD, and some HA20 cases exhibit varying degrees of perianal lesions, including severe perianal fistula. A rare disease study identified a 187 kb *de novo* microdeletion in the *TNFAIP3* gene in an infant with infantile-onset IBD and perianal lesions, leading to a diagnosis of HA20 (32). Therefore, HA20 should be differentiated from infantile-onset IBD with perianal lesions, and CNV analysis should be considered alongside whole exome sequencing (WES) to assess *TNFAIP3* loss (33). This approach provides more comprehensive genetic information for accurate diagnosis and treatment. Familial Mediterranean fever (FMF), caused by biallelic variants in exon 10 of the *MEFV* gene, is characterized by recurrent fever, abdominal pain, and joint inflammation. Although HA20 and FMF share clinical similarities and both develop in early childhood, they are distinct autoinflammatory diseases (34, 35). Treatment strategies also overlap, with most FMF patients responding well to colchicine, while a minority require IL-1 blockers. Given that HA20 patients exhibit excessive production of pro-inflammatory cytokines (36–38), biologics targeting cytokines effectively inhibit systemic inflammatory responses, These include anti-TNF-α agents (infliximab, adalimumab), anti-IL-1 agents (anakinra, canakinumab), and anti-IL-6 agents (tocilizumab). Corticosteroids are routinely used for symptomatic control, and some patients respond to colchicine therapy, either alone or in combination with glucocorticoids or mesalazine (39). Other immunotherapies, such as anti-CD20 monoclonal antibodies (rituximab), JAK-1 and -3 inhibitors (tofacitinib), and thalidomide, have also been widely used (40–42). Hematopoietic stem cell transplantation can be considered for severe refractory cases.

All patients in this study received thalidomide therapy. The mechanism of thalidomide treatment for HA20 primarily involves the following aspects: First, inhibition of inflammatory factor release. Thalidomide reduces the production and release of pro-inflammatory cytokines such as TNF-α and IL-6 by suppressing the NF-κB signaling pathway. Second, regulation of immune cell function. Thalidomide modulates the balance of T cell subsets, promoting Th1 cell differentiation while inhibiting hyperactive Th2 and Th17 cells, thereby restoring immune homeostasis and aiding in the control of inflammatory responses. Third, inhibition of angiogenesis. Thalidomide reduces abnormal angiogenesis by suppressing the expression of angiogenic factors, thereby alleviating congestion and edema in inflammatory tissues and mitigating symptoms. In summary, thalidomide exerts its therapeutic effects on HA20 through its immunomodulatory, anti-inflammatory, and angiogenic-inhibiting properties.

HA20 is generally considered a chronic condition requiring ongoing management, with the majority of patients necessitating long-term or even lifelong treatment. This is due to the underlying cause of the disease—insufficient A20 protein function resulting from a TNFAIP3 gene mutation—which is congenital and persistent. These conditions also carry certain side effects. Common side effects of biologics include: secondary failure; acute or delayed infusion reactions (more frequently observed with infliximab); increased risk of infection; and potential minor elevations in the risk of malignancies, autoimmune phenomena (e.g., anti-nuclear antibodies), and demyelinating lesions with prolonged use. Common side effects of immunomodulators include: gastrointestinal intolerance and myelosuppression and hepatotoxicity associated with azathioprine. Common side effects of glucocorticoids include: Cushing’s syndrome, hypertension, hyperglycemia, osteoporosis, and increased risk of infection with prolonged or high-dose use. The risks of hematopoietic stem cell transplantation are high, including graft rejection, graft-versus-host disease, severe infections, and organ toxicity. In summary, the treatment of HA20 requires balancing effective inflammation control with safe management of drug side effects. Patients should adhere to medical advice and maintain regular follow-ups during long-term treatment.

Current medical research and standard clinical practice have not yet developed gene therapies targeting HA20. Existing treatments rely entirely on immunosuppressants and biologics to control symptoms. However, gene therapies targeting the *TNFAIP3* gene itself are being explored as cutting-edge technologies in other non-HA20 disease areas. These studies demonstrate that increasing A20 expression through specific techniques is theoretically feasible and shows therapeutic potential. Although these studies involve the A20 gene, they do not constitute a root-cause treatment for HA20.

The CNV in patient 3 has not been previously reported, though other cases have documented similar deletions or larger/smaller fragments. The deleted segment contains the *PEX7*, *IL20RA*, and *IL22RA2* genes. *PEX7* is a peroxisome-targeting signal receptor essential for maintaining peroxisomal function (43). This organelle plays a crucial role in multiple metabolic processes, particularly phospholipid metabolism during skeletal development. Deficiency of this gene leads to skeletal abnormalities, with complete deletion causing “root-and-shelf punctate chondrodysplasia,” a bone disorder. In large deletions, haploinsufficiency may manifest as mild skeletal abnormalities, growth retardation, and distinctive facial features. The *IL20RA* and *IL22RA2* genes, both members of the interleukin receptor family, are critical for immune regulation-particularly in skin and mucosal immunity-participating in maintaining skin barrier integrity and inflammatory responses. Their dysfunction results in dermatological conditions like atopic dermatitis, which was present in patient 3. Classic HA20 is characterized by fever, abdominal pain, diarrhea, rashes, oral ulcers and genital sores, intestinal ulcers, and arthritis. In patient 3, the observed arthritis may be associated with *PEX7* deficiency, while the absence of *IL20RA* and *IL22RA2* may exacerbate inflammatory dysregulation or rashes. Fumin Xue et al. have reported similar results (44).

In this study, mutations in patient 3 and patient 5 were novel and had not been previously reported. Although mutations in patients 1, 2, and 4 have been previously reported, there are still differences in clinical manifestations and treatment approaches. The mutations in patients 1 and 2 were also mentioned in the study by Xue et al. In this study, Patient 1 exhibited no genital ulcers but showed delayed motor and cognitive development. The treatment medications included: thalidomide, glucocorticoids, infliximab, and adalimumab. In contrast, in the study by Xue et al., patients with the same mutation presented genital ulcers but no delayed motor or cognitive development, with treatment medications consisting of thalidomide and infliximab. Patient 2 in this study had no liver injury and weakly positive autoantibodies, with treatment medications including thalidomide and glucocorticoids. In Xue et al. ‘s study, patients with the same mutation showed no liver injury and negative autoantibodies, with thalidomide as the treatment medication. Patient 4 in this study was a spontaneous mutation case, presenting with gastrointestinal lesions but no oral ulcers or rash, and was treated with thalidomide. In the case reported by Zhong et al., the patient’s mutation originated from a mother with Behçet’s disease, exhibiting oral ulcers and rash without gastrointestinal lesions, and was treated with a combination of hydroxychloroquine, prednisone, and cyclosporine. These findings demonstrate that the same mutation site can lead to different phenotypes (**Table 3**).

**Table 3:** Different characteristics of the same variant site.

variant site	c.866delA: p.H289Pfs* 3		c.1243_1247delAAAAC: p. N416Tfs* 11		c.133C>T: p. R45X	
Characteristics	Patient 1	The case reported by Xue et al	Patient 2	The case reported by Xue et al	Patient 3	The case reported by Zhong et al
Source of variant sites	*de novo*	*de novo*	*de novo*	*de novo*	*de novo*	mother
Recurrent fever	YES	YES	YES	YES	YES	YES
Oral ulcers	YES	YES	NO	NO	NO	YES
Genital ulcers	NO	YES	NO	NO	NO	NO
Skin lesions	NO	NO	YES	YES	NO	YES
Gastrointestinal lesions	YES	YES	YES	YES	YES	NO
Motor and cognitive development delay	YES	NO	NO	/	NO	/
ALT (0~40 U/L)	/	/	21.9	165.6	/	/
AST (0~40 U/L)	/	/	38	172.9	/	/
Autoantibodies	/	/	ANA (1∶100)	/	/	/
Treatment	Thalidomide, glucocorticoid, infliximab, adalimumab	Thalidomide, infliximab	Thalidomide, glucocorticoid	Thalidomide	Thalidomide, glucocorticoid	Hydroxychloroquine, prednisone, cyclosporine

MMA is a common type of organic acid metabolic disorder, categorized into early-onset and late-onset forms. In infancy, the early-onset type predominantly manifests with neurological symptoms such as seizures and motor dysfunction. Late-onset cases typically present with insidious onset and significant individual variations, with mild cases potentially remaining asymptomatic throughout life. Mutations in the *MMACHC* gene involved in patient 3 have been reported in the previous literature to be associated with late-onset MMA (45). L Lin et al. also documented such combinations in their research (46). Early genetic testing, diagnosis, and treatment are crucial for managing such complex diseases. Patients with both HA20 and MMA provide valuable clinical insights into HA20, although this case is not representative of the general pediatric population. HA20 exhibits highly variable clinical manifestations, making clinical diagnosis challenging. Gene sequencing is essential for definitive diagnosis.

HA20 has traditionally been considered a childhood-onset disease, but recent studies have revealed that a significant proportion of patients initially present with overt symptoms in adulthood. For instance, Harumi et al. described an adult-onset HA20 patient who was previously followed for Behcet’s disease and later developed rare central nervous system symptoms characteristic of HA20, which was subsequently confirmed by genetic testing. Adult-onset HA20 often manifests with delayed onset and diverse presentations, frequently leading to misdiagnosis as Behcet’s disease, Crohn’s disease, or other autoimmune inflammatory disorders. Therefore, for patients presenting with Behcet’s disease-like symptoms, recurrent fever, and systemic inflammation in adulthood, particularly those with poor response to conventional therapy, the possibility of HA20 should be considered, and *TNFAIP3* gene testing should be performed to establish a definitive diagnosis and guide targeted treatment. The treatment approach is similar to that for pediatric patients, but adult comorbidities (e.g., hypertension, diabetes, etc.) must be taken into account.

However, this study still has limitations. This study also has limitations. Firstly, the small sample size may not cover all phenotypes of HA20. Secondly, the newly identified variants were classified solely based on the ACMG criteria without functional validation, and future research should be more comprehensive. Future research directions should include: establishing multicenter, large-sample cohorts of HA20 patients to conduct more reliable genotype-phenotype association analyses and conducting prospective studies to compare the long-term efficacy and safety of different biologics as first-line treatments, ultimately forming evidence-based diagnostic and therapeutic consensus. Through a systematic analysis of five HA20 patients, this study provides a complete clinical atlas of this rare autoimmune inflammatory disease, covering genetic diagnosis, clinical phenotypes, endoscopic features, and treatment responses. For the first time, this study reports two novel pathogenic mutations located in the *TNFAIP3* gene: NC_000006.11: g.136693638_138817508del and c.1903_1906delAAAC: p. K635fs* 61. The discovery of these new mutations not only enriches the genetic mutation database of HA20 but also underscores the necessity of genetic testing for patients with atypical symptoms. Through detailed multidimensional clinical data, this study emphasizes the critical importance of early genetic diagnosis for HA20. The reported new mutations expand disease understanding, while individualized management strategies based on endoscopic features and targeted therapies significantly improve patient outcomes. Despite the increasing global reports of HA20 cases in recent years, this study adds detailed cohort data from the China population, providing a comprehensive case analysis from genetic testing to corresponding clinical and endoscopic phenotypes, and finally to specific treatment responses. This study reinforces the paradigm of precision medicine in the management of rare diseases.

## Data Availability

The original contributions presented in the study are included in the article/**Supplementary Material**. Further inquiries can be directed to the corresponding author.
