# Supplementary material for: Five children with haploinsufficiency of A20 caused by heterozygous mutations in the TNFAIP3 gene
Source: Front Immunol. 2026 Mar 11;17:1738656. doi: 10.3389/fimmu.2026.1738656 (PMC13013434; doi:10.3389/fimmu.2026.1738656)
Supplement: Supplementary file 1 [file Table1.docx]

The design was performed using the Primer-BLAST online tool (https://www.ncbi.nlm.nih.gov/tools/primer-blast/), where the forward primer sequence is, and the reverse primer sequence is. The amplified product length is. The PCR products were then processed by Shanghai Shenggong Bioengineering Co., Ltd. Zhengzhou Branch for first-generation sequencing.

c.133C>T: p. R45X

|  | **Sequence (5'->3')** | **Template strand** | **Length** | **Start** | **Stop** | **Tm** | **GC%** | **Self complementarity** | **Self 3' complementarity** |
| --- | --- | --- | --- | --- | --- | --- | --- | --- | --- |
| **Forward primer** | TGAGGGGAGGAGCTACAGAC | Plus | 20 | 409 | 428 | 60.03 | 60.00 | 4.00 | 1.00 |
| **Reverse primer** | CAAGCCTCAATGTGCTCTGC | Minus | 20 | 1404 | 1385 | 59.83 | 55.00 | 3.00 | 2.00 |
| **Product length** | 996 | | | | | | | | |


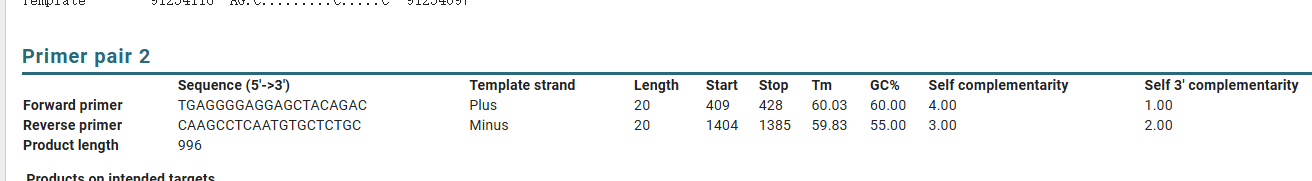


**Heat cycle parameters (the key is the choice of annealing temperature and extension time)**

1. ****Pre-transformation****：95℃，5 min (Complete denaturation of the template and activation of Taq enzyme)。
2. ****Cycling phase (30-35 cycles)****：
   - Variation: 95℃,30s (to unwind the double-stranded DNA);
   - Annealing: 58℃,30s
3. Annealing: 58℃,30s
4. ****Final extension: 72℃,5-10 min (to ensure complete extension of all products).****
5. ****Temperature Control: 4°C, ∞ (to prevent product degradation)****

c.866delA: p.H289Pfs* 3

## **Primer pair 1**

|  | **Sequence (5'->3')** | **Template strand** | **Length** | **Start** | **Stop** | **Tm** | **GC%** | **Self complementarity** | **Self 3' complementarity** |
| --- | --- | --- | --- | --- | --- | --- | --- | --- | --- |
| **Forward primer** | CCTCATGTGGAATAAGCACTGT | Plus | 22 | 397 | 418 | 58.39 | 45.45 | 6.00 | 3.00 |
| **Reverse primer** | TCTGCAATTCTGACTTTTGGGA | Minus | 22 | 1289 | 1268 | 58.17 | 40.91 | 5.00 | 0.00 |
| **Product length** | 893 | | | | | | | | |


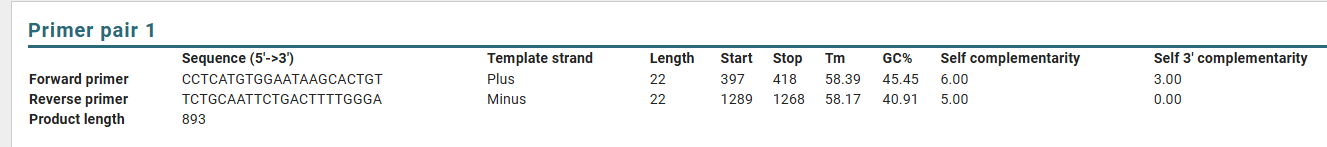


****Pre-transformation****: 95℃,5 min action: make the template DNA completely denature into single strand, and activate Taq enzyme.

****Cycling phase (30-35 cycles)****：

- - Variation: 95℃,30s function: to unwind the double-stranded DNA into single strands.
  - Annealing: 56℃,30s

1. Extension: 72℃,1 min basis: the product length is ~1 kb, according to the "1 min /kb" principle (to ensure that long fragments are fully extended).

****Final extension****：72℃,5-10 min action: ensure that all amplified products are fully extended.

****Temperature Control****：4℃,∞ (preserve the product and prevent degradation)

c.1903_1906delAAAC:
p. K635fs* 61

## **Primer pair 1**

|  | **Sequence (5'->3')** | **Template strand** | **Length** | **Start** | **Stop** | **Tm** | **GC%** | **Self complementarity** | **Self 3' complementarity** |
| --- | --- | --- | --- | --- | --- | --- | --- | --- | --- |
| **Forward primer** | GTCAGGACCTACCGTGCTTT | Plus | 20 | 104 | 123 | 59.68 | 55.00 | 5.00 | 0.00 |
| **Reverse primer** | TGGAGGTAGCATTTCGGACC | Minus | 20 | 1093 | 1074 | 59.46 | 55.00 | 3.00 | 3.00 |
| **Product length** | 990 | | | | | | | | |


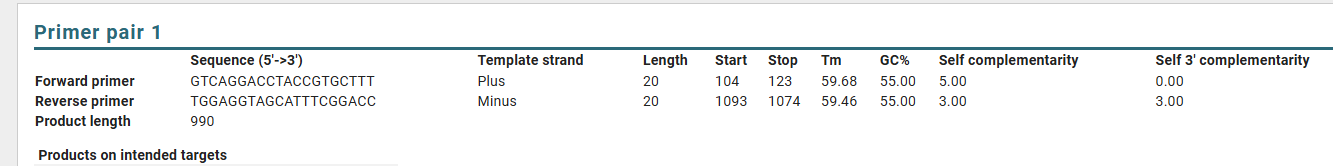


****Pre-transformation****：95℃,5 min effect: make the template DNA completely denature into single strand, and activate Taq enzyme.

****Cycling phase (30-35 cycles)****：

- - Variation: 95℃,30s function: to unwind the double-stranded DNA into single strands.

Annealing: 58℃,30s extension: 72℃,1 min basis: product length ~ 1 kb, Taq enzyme extension rate about 1 kb/min to ensure full extension of long fragments.

****Final extension****：72℃,5-10 min action: ensure that all amplified products are fully extended.

****Temperature Control****：4℃,∞ (preserve the product and prevent degradation)

c.1243_1247delAAAAC: p. N416Tfs* 11

## **Primer pair 1**

|  | **Sequence (5'->3')** | **Template strand** | **Length** | **Start** | **Stop** | **Tm** | **GC%** | **Self complementarity** | **Self 3' complementarity** |
| --- | --- | --- | --- | --- | --- | --- | --- | --- | --- |
| **Forward primer** | TGTGTCAGATCATGTTGCGTG | Plus | 21 | 515 | 535 | 59.20 | 47.62 | 4.00 | 2.00 |
| **Reverse primer** | TGACTTGGAACGCTGGTGAC | Minus | 20 | 1404 | 1385 | 60.53 | 55.00 | 3.00 | 3.00 |
| **Product length** | 890 | | | | | | | | |


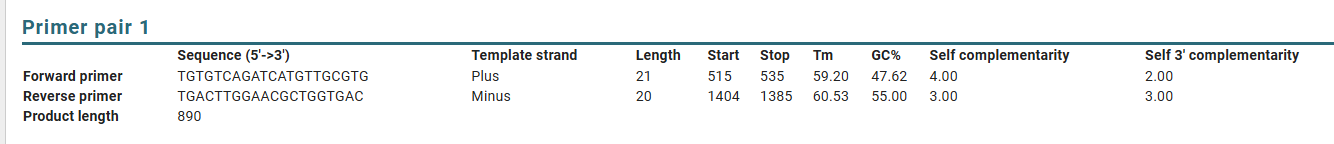


****Pre-transformation****：95℃,5 min effect: make the template DNA completely denature into single strand, and activate Taq enzyme.

****Cycling phase (30-35 cycles)****：

- - Variation: 95℃,30s function: to unwind the double-stranded DNA into single strands.

1. Annealing: 57℃ extension: 72℃,1 min basis: product length ~ 1 kb, Taq enzyme extension rate is about 1 kb/min, ensure that the long fragment is fully extended.

****Final extension****：72℃,5-10 min action: ensure that all amplified products are fully extended.

****Temperature Control****：4℃,∞ (preserve the product and prevent degradation)
